# Metamorphopsia associated with central retinal vein occlusion

**DOI:** 10.1371/journal.pone.0186737

**Published:** 2017-10-19

**Authors:** Koichiro Manabe, Rie Osaka, Yuki Nakano, Yukari Takasago, Tomoyoshi Fujita, Chieko Shiragami, Kazuyuki Hirooka, Yuki Muraoka, Akitaka Tsujikawa

**Affiliations:** 1 Department of Ophthalmology, Kagawa University Faculty of Medicine, Miki, Japan; 2 Department of Ophthalmology and Visual Sciences, Kyoto University Graduate School of Medicine, Kyoto, Japan; Bascom Palmer Eye Institute, UNITED STATES

## Abstract

This prospective study aimed to investigate metamorphopsia in eyes with central retinal vein occlusion (CRVO) and included 28 eyes (28 patients) with unilateral CRVO that had macular edema (ME) in the acute phase. The ME was treated with anti-vascular endothelial growth factor agents. At baseline and at 1 and 6 months after initiation of treatment, quantitative measurements of metamorphopsia were performed using M-CHARTS and the retinal morphologic changes were examined by optical coherence tomography. At baseline, metamorphopsia was detected on M-CHARTS in 14 (50.0%) eyes. The mean M-CHARTS score was 0.37 ± 0.53. At 1 month and 6 months after initiation of treatment, there was substantial resolution of ME and significant recovery of visual acuity. In contrast, metamorphopsia was still detected in 16 eyes at 6 months; the mean M-CHARTS scores were 0.29 ± 0.37 at 1 month and 0.32 ± 0.38 at 6 months, and had not significantly improved from baseline (p = 0.580, and p = 0.604, respectively). Although the M-CHARTS score at 6 months was associated with the baseline M-CHARTS score (p = 0.004), it did not have any associations with morphologic parameters at baseline. However, the M-CHARTS score at 6 months was significantly associated with foveal photoreceptor status, height of serous detachment, and parafoveal thickening at 1 month. Metamorphopsia associated with CRVO could be quantified using M-CHARTS, and often persisted in contrast with the recovery of visual acuity and resolution of ME after treatment with anti-vascular endothelial growth factor agents.

## Introduction

Macular edema (ME) is one of the most vision-threatening complications associated with acute retinal vein occlusion (RVO) [1, 2]. Although the introduction of anti-vascular endothelial growth factor (VEGF) agents has improved the visual prognosis of RVO [3, 4], patients with this condition often suffer from symptomatic metamorphopsia even when impairment of visual acuity (VA) and ME are substantially ameliorated [5]. Metamorphopsia would certainly degrade the quality of vision [6–11].

Thus far, our understanding of metamorphopsia associated with RVO remains limited due to a lack of methodologies to quantify the degree of metamorphopsia [5]. The traditional Amsler grid chart is a convenient method for detecting metamorphopsia but does not allow quantitative measurements [12]. The M-CHARTS developed by Matsumoto et al. [13] enable quantitative evaluation of the degree of metamorphopsia. Recently, our group (Manabe et al. [14]) applied M-CHARTS in 42 eyes with acute branch retinal vein occlusion (BRVO) and reported that metamorphopsia was detected on M-CHARTS in 29 (69.0%) eyes with acute BRVO and that it was usually persistent even after regression of ME in response to treatment with anti-VEGF agents.

Retinal involvement in central retinal vein occlusion (CRVO) is more extensive than in BRVO and is often accompanied by more severe morphologic and functional impairments [2, 15, 16]. However, it is unclear whether the severe pathologies in CRVO would cause more or less symptomatic metamorphopsia. To date, no information has been available on the clinical characteristics of CRVO-associated metamorphopsia, including the degree, prevalence, and prognosis. The aim of this study was to acquire quantitative measurements of metamorphopsia using M-CHARTS in eyes with CRVO in order to obtain this information.

## Patients and methods

The study was approved by the Ethics Committee at Kagawa University Faculty of Medicine and conducted in accordance with the tenets of the Declaration of Helsinki. Written informed consent was obtained from each subject before any study procedures or examinations were performed.

### Patients

This prospective study enrolled 28 consecutive patients with unilateral CRVO who had ME in the acute phase and were examined at the Department of Ophthalmology in Kagawa University Hospital between September 2014 and April 2016.

The inclusion criteria were: (1) symptomatic CRVO, in which the retinal edema and hemorrhage involved the macula, (2) a foveal thickness >250 μm at the initial visit as measured by optical coherence tomography (OCT), and (3) symptoms of less than 3 months’ duration prior to the baseline examination. The diagnosis of CRVO was based on fundus examination and findings on fluorescein angiography. Ischemic CRVO was defined as a retinal nonperfusion area of more than 10 disc diameters on fluorescein angiography. Eyes with BRVO and hemi-CRVO were not included. Eyes with co-existing ocular disease (age-related macular degeneration, retinitis pigmentosa, diabetic retinopathy, or retinal macroaneurysm) were excluded. The diagnosis of CRVO and exclusion of other retinal diseases were based on the findings of fundus examination, OCT, and fluorescein angiography as determined by 3 retina specialists (KM, RO, and AT). Patients with a history of intervention for ME were also excluded.

### Schedule of treatment and evaluation

A medical history was obtained from each patient at the initial visit. All patients underwent a comprehensive ophthalmologic examination, including measurement of best-corrected VA using the Landolt chart and the degree of metamorphopsia by M-CHARTS (Inami, Tokyo, Japan), determination of intraocular pressure, indirect ophthalmoscopy, slit-lamp biomicroscopy with a noncontact lens, OCT examinations (Spectralis HRA+OCT; Heidelberg Engineering, Heidelberg, Germany), and fluorescein angiography (Optos 200Tx imaging system, Optos PLC, Dunfermline, UK). Each patient was scheduled for re-evaluation of retinal morphology and visual function every month. Measurements of best-corrected VA and intraocular pressure as well as OCT examinations were performed at every visit. Fluorescein angiography was performed if deemed necessary. M-CHARTS measurements were performed at baseline and at 1 month and 6 months after the initial anti-VEGF injections.

Throughout the observation period after baseline, each patient was treated for ME by intravitreal injection of the same anti-VEGF agent. Ranibizumab (Lucentis; Novartis Pharma, Tokyo, Japan) was used in 9 eyes and aflibercept (Eylea; Bayer, Osaka, Japan) was used in 19 eyes. Each eye was examined every month after the initial treatment and pro re nata (PRN) injections were performed when ME or serous retinal detachment was evident at the fovea on OCT sections. None of the eyes received another treatment for ME, such as grid laser photocoagulation, steroid treatment, or surgical intervention. Panretinal photocoagulation was performed for eyes with ischemic CRVO when ocular neovascularization was recognized or its presence was strongly suspected.

### Evaluation of metamorphopsia

Commercially available M-CHARTS were used for quantitative measurement of the metamorphopsia. The principle of M-CHARTS has been described in detail previously [13, 14]. In brief, an M-CHART comprises a series of 19 dotted line tests. In each chart, the intervals of each dot range from 0.2° to 2.0°. A fixation point is printed in the center of each line, measuring 0.3° of the visual angle. First, an examiner presents a chart with a solid line at a distance of 30 cm under correction of refraction, followed by charts with dotted lines of incrementally increasing spacing. For each chart, the patient is asked to state whether the presented line is distorted or not. As the visual angle increases, the degree of metamorphopsia decreases. When the patient recognizes the presented line as being straight, the visual angle of that line is taken as the degree of metamorphopsia. M-CHARTS were presented to the patient in a vertical direction and then in a horizontal direction. The vertical and horizontal scores were measured, and the higher score was used as the M-CHARTS score for the eye. This measurement was performed in each patient at baseline and at 1 and 6 months after the initial treatment.

### Measurement of structural changes in the retina using OCT

Morphologic changes associated with CRVO were quantitatively evaluated by OCT, as described previously [14]. The entire macular area was examined with sequential OCT sectioning to detect any serous retinal detachment or cystoid spaces.

**Fig 1:**
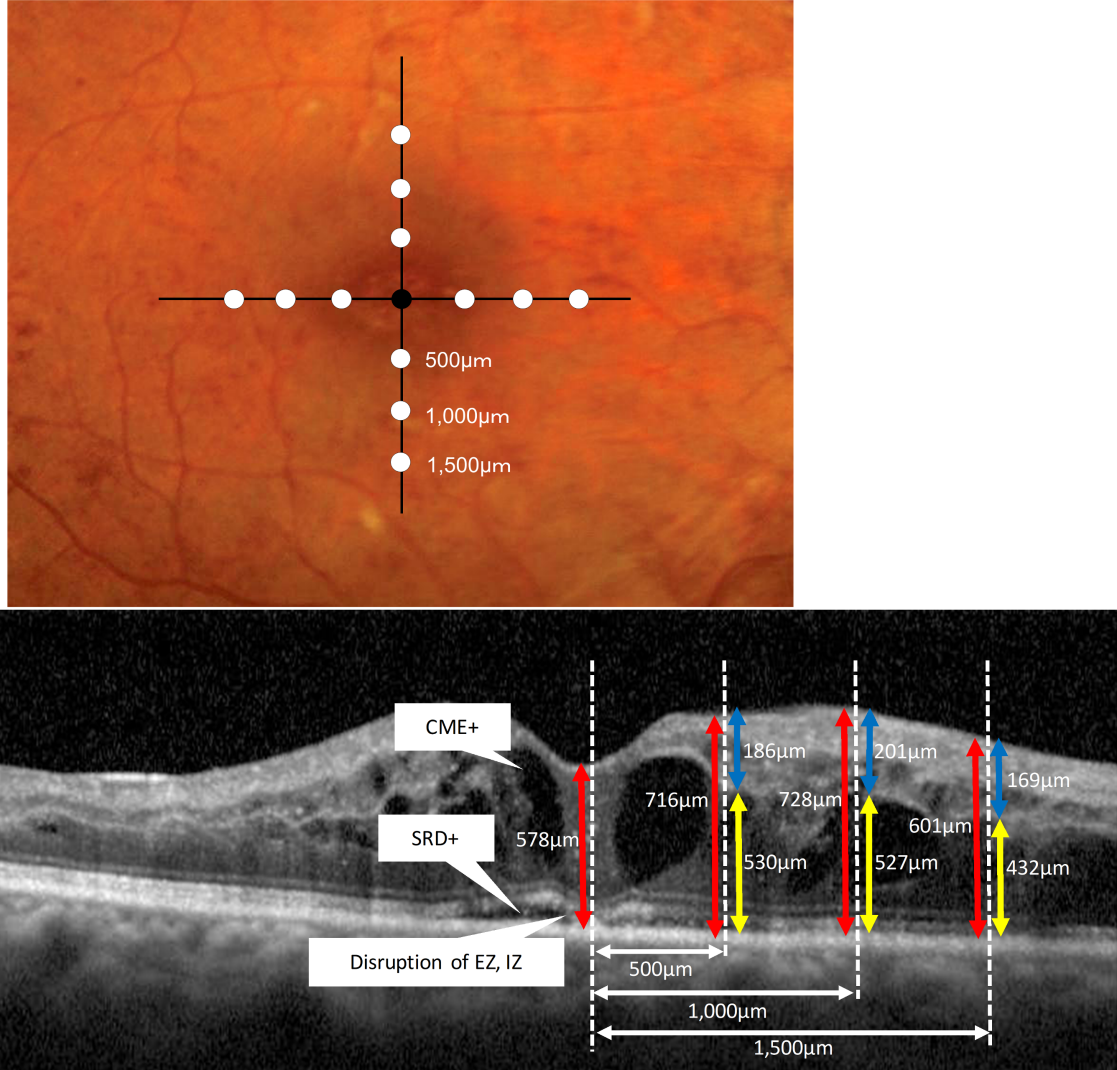
Quantitative measurements of retinal morphologic changes associated with acute central retinal vein occlusion using optical coherence tomography. On the horizontal and vertical sections through the foveal center, the inner (blue arrows), outer (yellow arrows), and total (red arrows) retinal thickness was measured at 0.5 mm, 1.0 mm, and 1.5 mm from the center of the fovea towards both sides of the retina, respectively. The maximum thickness of the inner, outer, or total retina was determined as the largest of the 12 measurements (white dot). The total foveal thickness was defined as the distance between the vitreoretinal interface and the inner surface of the retinal pigment epithelium (black dot).

Quantitative measurements were performed using vertical and horizontal sections acquired through the foveal center. The thickness of the inner retina was defined as the vertical distance between the vitreoretinal interface and the outer surface of the inner nuclear layer. The thickness of the outer retina was defined as the vertical distance between the outer surface of the inner nuclear layer and the inner surface of the retinal pigment epithelium. The total retinal thickness was defined as the distance between the vitreoretinal interface and the inner surface of the retinal pigment epithelium (Fig 1).

On the vertical and horizontal sections through the foveal center, the inner, outer, and total retinal thickness was measured at 0.5 mm, 1.0 mm, and 1.5 mm from the foveal center, respectively (Fig 1) [14]. The maximum thickness of the inner, outer, or total retina was defined as the maximum value among these 12 measurements. The thickness of the serous retinal detachment was measured manually at the largest point, which was usually the fovea [17]. These measurements were performed by a masked grader at the initial visit and at 1 month and 6 months after the initial treatment.

A disruption of the ellipsoid or interdigitation zone band was diagnosed when a loss and irregularity of each hyper-reflective line was detected in the 1 mm foveal area. This evaluation at baseline was sometimes difficult because of the marked ME and dense retinal hemorrhage. This evaluation was performed at 1 month and 6 months after the initial treatment by a masked grader.

### Statistical analysis

The statistical analysis was performed using SPSS version 23.0.0 software (IBM Japan, Tokyo, Japan). All values are presented as the mean ± standard deviation. The best-corrected VA was converted to a logarithm of the minimum angle of resolution (logMAR) equivalent for statistical analysis. Comparisons between baseline and post-treatment values were performed using the paired *t*-test. Bivariate relationships were analyzed using Pearson’s correlation coefficient to evaluate the correlation between each measurement value and the M-CHARTS score. A p-value < 0.05 was considered to be statistically significant.

## Results

Table 1 shows the baseline measurements for all 28 patients (19 men, 9 women) enrolled in the study. The mean patient age was 70.3 ± 10.6 years and the mean duration of symptoms was 2.3 ± 2.1 weeks. At baseline, all eyes showed decreased VA and ME associated with acute CRVO; the mean total foveal thickness was 662.1 ± 278.9 μm and the mean VA in logMAR was 0.70 ± 0.41. Twenty (71.4%) of the 28 eyes showed serous retinal detachment at the fovea and 24 (85.7%) showed cystoid ME. Due to the severe ME and retinal hemorrhage, it is often difficult to determine the integrity of the ellipsoid or interdigitation zone at baseline. At baseline, 21 eyes had non-ischemic CRVO and 7 eyes had ischemic CRVO.

**Table 1:** Measurements in eyes with central retinal vein occlusion at baseline and at 1 month and 6 months after initial treatment.

	Baseline	1 month	p-value	6 months	p-value
Visual acuity, logMAR	0.70 ± 0.41	0.46 ± 0.33	< 0.001	0.44 ± 0.45	0.007
Total foveal thickness, μm	662.1 ± 278.9	219.3 ± 90.4	< 0.001	277.2 ± 168.9	< 0.001
Thickness of serous retinal detachment, μm	145.2 ± 181.9	18.3 ± 63.3	0.001	16.9 ± 47.3	0.722
Maximum of total retinal thickness, μm	686.6 ± 210.2	409.8 ± 155.0	< 0.001	417.7 ± 133.9	< 0.001
Maximum of inner retinal thickness, μm	284.8 ± 120.4	205.6 ± 44.0	0.002	205.6 ± 41.1	0.002
Maximum of outer retinal thickness, μm	501.1 ± 186.4	238.2 ± 155.6	< 0.001	251.7 ± 119.5	< 0.001
Disruption of ellipsoid zone band, n	-	17		17	
Disruption of interdigitation zone band, n	-	14		15	
M-CHARTS					
Vertical score	0.32 ± 0.48	0.26 ± 0.38	0.471	0.28 ± 0.34	0.684
Horizontal score	0.29 ± 0.50	0.23 ± 0.34	0.570	0.24 ± 0.36	0.592
M-CHARTS score (higher score)	0.37 ± 0.53	0.29 ± 0.37	0.580	0.32 ± 0.38	0.604

Visual acuity is converted to a logarithm of the minimum angle of resolution (logMAR) equivalent. The M-CHARTS score is the higher score of the vertical and horizontal scores of M-CHARTS. p-value, compared with baseline values

Quantitative measurement of metamorphopsia was performed using M-CHARTS. Metamorphopsia was detected in the vertical and/or horizontal directions in 14 (50.0%) eyes. The mean vertical and horizontal scores were 0.32 ± 0.48 and 0.29 ± 0.50, respectively. The mean higher score of the vertical and horizontal scores was 0.37 ± 0.53.

Each patient was treated with intravitreal anti-VEGF injections (ranibizumab in 9 eyes, aflibercept in 19 eyes). The mean number of injections was 2.5 ± 1.1 (range 1–6). One month after the initial treatment, most eyes showed a substantial reduction of ME. The total foveal thickness was significantly decreased to the physiologic level (219.3 ± 90.4 μm, p < 0.001) and VA was improved to 0.46 ± 0.33 (p < 0.001). However, the parafoveal retinal thickening was often persistent at 1 month. The maximum inner, outer, and total retinal thicknesses were significantly decreased when compared with those at baseline (p < 0.002) but were still greater than physiologic values. With additional PRN injections, each eye could maintain the initial recovery of VA for 6 months (0.44 ± 0.45, p = 0.020).

In contrast with the improvement of ME and VA, metamorphopsia was still detected in 12 eyes at 1 month. Six months after the initial treatment, metamorphopsia was still detected in 16 eyes; at this time, metamorphopsia had completely resolved in 4 of 14 eyes that had metamorphopsia at baseline. Metamorphopsia was newly recognized in 6 eyes in the course of treatment. At 1 month and 6 months, the mean M-CHARTS scores did not decrease significantly when compared with baseline values (0.29 ± 0.37, p = 0.580 at 1 month; 0.32 ± 0.38, p = 0.604 at 6 months).

Table 2 shows the correlation between response to treatment and M-CHARTS scores in patients subgrouped according to whether they had ischemic or non-ischemic CRVO. We found a significant difference in visual acuity between the groups at 1 month and 6 months. However, we did not find a significant difference in M-CHARTS scores or other retinal morphologic measurements at these time points.

**Table 2:** Subgroup analysis of correlation between response to treatment and M-CHARTS scores in patients with ischemic CRVO and patients with non-ischemic CRVO.

	Baseline			1 month			6 months		
	Ischemic CRVO	Non-ischemic CRVO	p-value	Ischemic CRVO	Non-ischemic CRVO	p-value	Ischemic CRVO	Non-ischemic CRVO	p-value
Visual acuity, logMAR	0.627 ± 0.390	0.63 ± 0.39	0.14	0.77 ± 0.41	0.36 ± 0.29	0.01	0.89 ± 0.38	0.29 ± 0.36	<0.01
Total foveal thickness, μm	760.6 ± 311.4	619.2 ± 253.2	0.17	214.6 ± 113.0	220.8 ± 81.5	0.88	262.7 ± 171.8	282.0 ± 167.6	0.80
Thickness of serous retinal detachment, μm	298.0 ± 211.2	94.3 ± 137.3	0.35	12.6 ± 30.8	20.1 ± 70.8	0.79	127.0 ± 73.0	93.0 ± 52.0	0.23
Maximum of total retinal thickness, μm	822.9 ± 208.4	641.2 ± 190.2	0.05	403.3 ± 91.1	412.0 ± 171.0	0.90	427.4 ± 145.2	414.4 ± 129.8	0.83
Maximum of inner retinal thickness, μm	359.4 ± 182.8	259.9 ± 75.5	0.06	207.6 ± 22.4	204.6 ± 49.1	0.88	211.9 ± 31.0	203.5 ± 43.8	0.66
Maximum of outer retinal thickness, μm	598.1 ± 132.8	468.8 ± 190.5	0.12	237.4 ± 109.0	238.4 ± 168.3	0.99	253.6 ± 128.7	251.1 ± 116.2	0.96
Disruption of ellipsoid zone band, n	-	-		3	14		1	16	
Disruption of interdigitation zone band, n	-	-		3	11		1	14	
M-CHARTS									
Vertical score	0.04 ± 0.10	0.42 ± 0.52	0.08	0.23 ± 0.30	0.28 ± 0.40	0.78	0.24 ± 0.18	0.30 ± 0.38	0.73
Horizontal score	0.06 ± 0.09	0.36 ± 0.56	0.16	0.14 ± 0.23	0.26 ± 0.37	0.44	0.13 ± 0.21	0.28 ± 0.40	0.35
M-CHARTS score (higher score)	0.07 ± 0.12	0.46 ± 0.57	0.09	0.30 ± 0.32	0.29 ± 0.40	0.94	0.27 ± 0.18	0.34 ± 0.41	0.70

Visual acuity is converted to the logMAR equivalent. The M-CHARTS score is the higher score among the vertical and horizontal scores of M-CHARTS. p-value, compared with baseline values. CRVO, central retinal vein occlusion; logMAR, logarithm of the minimum angle of resolution.

Table 3 shows the association between M-CHARTS scores and retinal morphologic parameters on OCT obtained at the same evaluation points (baseline, 1 month, and 6 months after initial treatment). At baseline, the M-CHART score did not have any association with morphologic OCT parameters. At 1 month and 6 months, post-treatment M-CHART scores showed no association with age, VA, total foveal thickness, or retinal thickness of each layer; however, the post-treatment metamorphopsia (at 1 and 6 months) showed associations with the condition of the outer aspects of the foveal photoreceptor layer; the post-treatment M-CHARTS scores were closely correlated with disruptions of the ellipsoid and interdigitation zones at 1 month and 6 months, respectively (Table 2).

**Table 3:** Associations between M-CHARTS scores and other measurements at the same time points (baseline and at 1 month and 6 months after initial treatment).

	Baseline		1 month		6 months	
	r	p-value	r	p-value	r	p-value
Age	0.04	0.823	0.25	0.207	-0.05	0.812
Visual acuity in logMAR	0.02	0.911	0.26	0.191	0.03	0.882
Total foveal thickness	0.25	0.209	0.09	0.663	-0.07	0.720
Thickness of serous retinal detachment	0.01	0.971	0.09	0.572	-0.86	0.143
Maximum of total retinal thickness	0.26	0.189	0.08	0.673	-0.01	0.961
Maximum of inner retinal thickness	0.05	0.807	0.16	0.404	0.10	0.608
Maximum of outer retinal thickness	0.30	0.126	0.09	0.639	-0.05	0.819
Serous retinal detachment	0.26	0.179	0.09	0.645	0.09	0.666
Cystoid macular edema	0.17	0.390	0.04	0.836	-0.04	0.848
Disruption of ellipsoid zone band	-	-	0.57	0.001	0.40	0.003
Disruption of interdigitation zone band	-	-	0.57	0.002	0.61	<0.001

Visual acuity is converted to a logarithm of the minimum angle of resolution (logMAR) equivalent. M-CHARTS score is the higher score of the vertical and horizontal scores of M-CHARTS.

**Fig 2:**
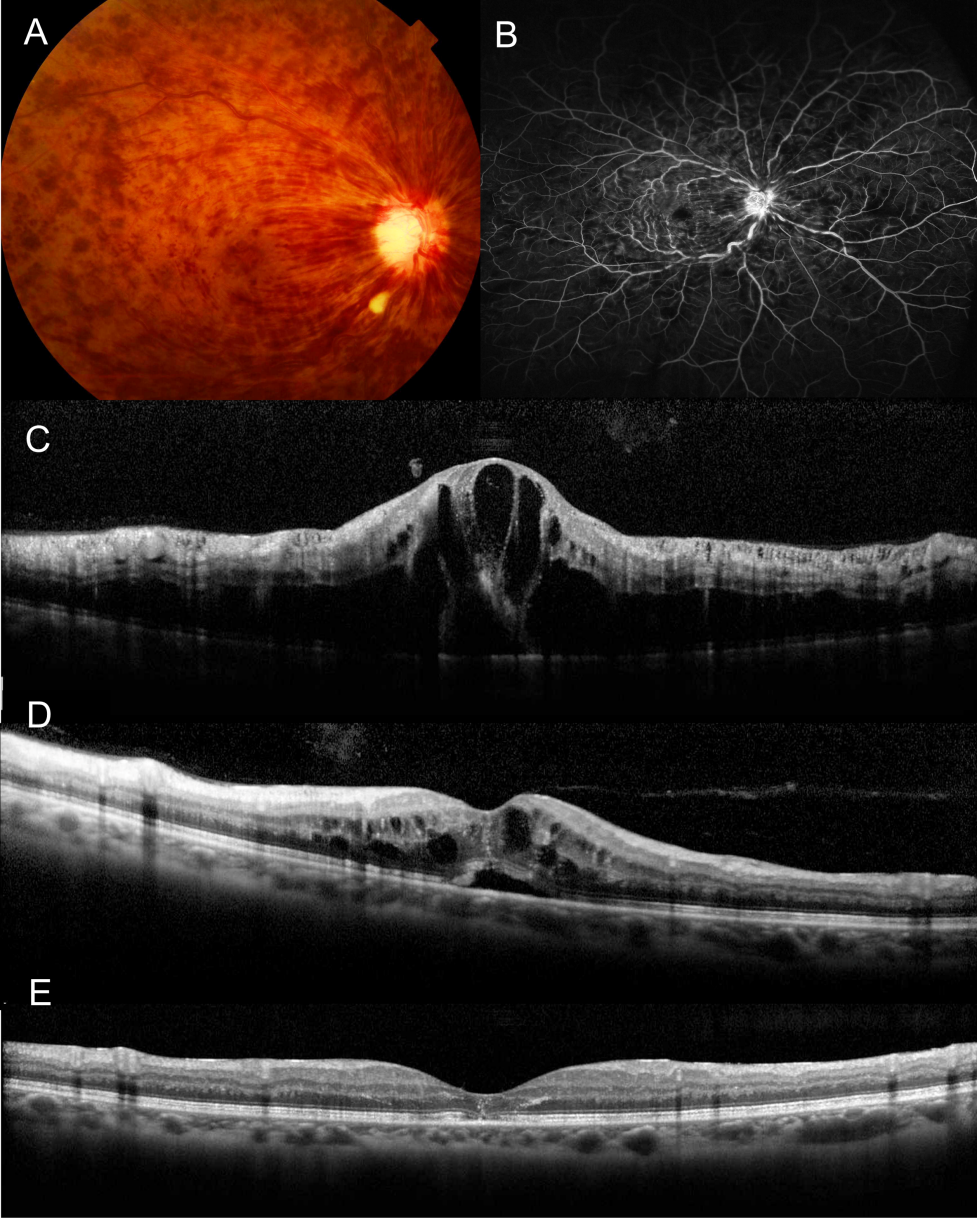
Persistent metamorphopsia after resolution of macular edema associated with acute CRVO. A 47-year-old man had visual disturbance due to acute CRVO in the right eye. (**A**) Fundus photograph at baseline. (**B**) Fluorescein angiogram at baseline. (**C**) The baseline vertical section of an optical coherence tomography (OCT) scan through the foveal center shows the foveal cystoid spaces and retinal thickening in the affected retina. The visual acuity of the left eye was 1.00 in logMAR. M-CHARTS scores were 1.5. The eye was treated with an intravitreal injection of aflibercept. (**D**) One month after the initial injection, a vertical OCT section shows reduction of the macular edema. The visual acuity was 0.82 in logMAR and M-CHARTS scores were still 0.5. (**E**) Six months after the initiation of treatment, a vertical OCT section shows complete absorption of the macular edema. The visual acuity was improved to 0.40 in logMAR. However, M-CHARTS scores were still 0.4. Abbreviations: CRVO, central retinal vein occlusion; OCT, optical coherence tomography.

Table 4 shows the association between the post-treatment M-CHARTS score at 6 months and the measurements at baseline and 1 month after the initial treatment. Although the M-CHARTS score at 6 months correlated with the baseline M-CHARTS score (p = 0.004), it did not have any association with the baseline morphologic parameters. However, the M-CHART score at 6 months was associated with morphologic changes at 1 month; this score showed significant associations with foveal photoreceptor status (p < 0.001 for disruption of the ellipsoid zone band and p = 0.007 for disruption of the interdigitation zone band), thickness of serous retinal detachment (p = 0.004), and the maximum of inner (p = 0.016), outer (p = 0.009), or total (p = 0.010) retinal thickness in the parafovea. In addition to the morphologic changes at 1 month, the M-CHARTS score at 1 month showed a close correlation with the final M-CHARTS score (p < 0.001). All 12 eyes that had metamorphopsia at 1 month had persistent metamorphopsia at 6 months (Fig 2).

**Table 4:** Association between post-treatment (6-month) m-charts score and measurements obtained at baseline and at 1 month after initial treatment.

	Baseline		1 month	
	r	p-value	r	p-value
Visual acuity	0.04	0.857	0.28	0.149
Total foveal thickness	0.16	0.415	-0.16	0.431
Thickness of serous retinal detachment	0.13	0.495	0.53	0.004
Maximum of total retinal thickness	0.17	0.395	0.48	0.010
Maximum of inner retinal thickness	0.16	0.423	0.45	0.016
Maximum of outer retinal thickness	0.08	0.681	0.49	0.009
Serous retinal detachment	0.16	0.409	0.33	0.086
Cystoid macular edema	-0.22	0.258	0.16	0.432
Disruption of ellipsoid zone band	-	-	0.60	<0.001
Disruption of interdigitation zone band	-	-	0.50	0.007
M-CHARTS score	0.52	0.004	0.72	<0.001

Visual acuity is converted to a logarithm of the minimum angle of resolution (logMAR) equivalent.

M-CHARTS score is the higher score of the vertical and horizontal scores of M-CHARTS.

## Discussion

Thus far, several investigators have used M-CHARTS for quantitative measurement of metamorphopsia associated with RVO. Murakami et al. [18] reported metamorphopsia in 28 (93%) of 30 eyes with cystoid ME associated with BRVO. Recently, our group (Manabe et al. [14]) reported metamorphopsia in 29 (69.0%) of 42 eyes with acute BRVO. Nakagawa et al. [7] and Achiron et al. [5] reported that metamorphopsia did not regress with treatment in eyes with BRVO. Manabe et al. [14] reported that only 3 (10.3%) of 29 eyes with metamorphopsia at baseline achieved complete resolution at 1 month after anti-VEGF therapy. Judging from previous reports, the prevalence of metamorphopsia is quite high in eyes with RVO, and once metamorphopsia has developed, it often persists even after resolution of ME.

In the recent report on eyes with BRVO by Manabe et al. [14], the mean total foveal thickness was 467.2 ± 191.5 μm and the mean VA in logMAR was 0.33 ± 0.31, but the mean M-CHARTS score was 0.68 ± 0.67. In the current study, mean total foveal thickness was 662.1 ± 278.9 μm and mean VA in logMAR was 0.70 ± 0.41. However, the mean M-CHARTS score was 0.37 ± 0.53. Although the severity of ME seems to be greater in CRVO, the prevalence and degree of metamorphopsia was unexpectedly higher in BRVO. The reason for this is unclear. Murakami et al. [18] hypothesized that because the lesion is located either above or below the fovea, the detection power tends to be stronger for vertical lines than for horizontal lines on M-CHARTS in eyes with BRVO. In contrast, a CRVO lesion tends to involve the entire retina. The finding that CRVO lesions are not located in one part of the retina could be explained by the fact that the degree of metamorphopsia in CRVO is lower than that in BRVO. Another possibility is that the relatively preserved VA in eyes with BRVO may be involved in symptomatic metamorphopsia.

The pathomorphology of metamorphopsia is still controversial [12]. Various studies using OCT have shown that the severity of metamorphopsia caused by epiretinal membrane (ERM) is primarily related to the thickness of the inner nuclear layer [19–21]. Okamoto et al. [20] speculated that structural changes in horizontal cells, bipolar cells, amacrine cells, and Müller cells would inhibit normal function of synaptic junctions and decrease photoreceptor sensitivity, causing metamorphopsia. Using fundus autofluorescence, Nitta et al. [22] also demonstrated horizontal movement of retinal vessels induced by ERM contraction. ERM contraction would induce non-uniform horizontal shift of photodetector cells [23]. In eyes with ERM, such partial disarray of photoreceptors in the macular area may contribute to metamorphopsia.

Similarly, Murakami et al. [18] reported that the severity of metamorphopsia in eyes with BRVO was associated with the presence of an inner retinal cyst. However, Manabe et al. [14] postulated that metamorphopsia from acute BRVO is mainly involved in the morphologic changes of the outer retina. In our patients with acute CRVO, none of the morphologic OCT parameters of the retina correlated with the M-CHARTS score. The mechanism of metamorphopsia is still unclear in eyes with RVO. We hypothesize that the horizontal retinal shift attributable to macular swelling and/or serous retinal detachment caused by the leakage produces disarray of the photoreceptor cells, leading to development of metamorphopsia [14, 23]. OCT could not show the horizontal shift of photoreceptor cells effectively. In our patients, the post-treatment M-CHARTS score was correlated with the integrity of the ellipsoid and interdigitation zones, and not with outer retinal thickness. Disruption of the ellipsoid and interdigitation zones may be a consequence of disarray of the outer segment of the macular photoreceptor cells due to the non-uniform retinal horizontal shift [24].

In our study, only 4 of 14 eyes that had metamorphopsia at baseline achieved complete resolution at 6 months. Similar to the previous reports on BRVO, metamorphopsia from CRVO tends to persist even after complete resolution of ME. In our patients with CRVO, the post-treatment M-CHARTS score showed a close correlation with the baseline M-CHARTS score. The disarray of the outer segment of photoreceptor cells that develops because of ME is difficult to resolve completely with treatment. Recently, adaptive optics-scanning laser ophthalmoscopy showed a disrupted cone mosaic arrangement in the parafoveal area in eyes with resolved BRVO [24]. Such disarray of the photoreceptors after absorption of ME may account for the persistent metamorphopsia in eyes with CRVO.

In the present study, none of the baseline morphologic parameters of the retina showed an association with the post-treatment (6-month) M-CHARTS score. However, some morphologic parameters at 1 month had an association with the post-treatment M-CHARTS score. Although total foveal thickness at 1 month showed no association with post-treatment M-CHARTS score, the maximum of the inner, outer, or total retinal thickness and thickness of serous retinal detachment at 1 month showed an association with the post-treatment (6-month) M-CHARTS score. After the initial injections, some eyes showed residual regional retinal thickening in the parafoveal area although foveal thickness was decreased to the physiologic level. Based on the current findings, such parafoveal thickening detected at 1 month may predict persistent or incurable metamorphopsia [25]. However, it is unclear whether this parafoveal thickening is derived from the regional horizontal retinal shift induced by ME or serous retinal detachment associated with CRVO.

One of the major limitations of this study is its small sample size. In addition, we evaluated metamorphopsia for 6 months after the initial treatment, which may not have been long enough to evaluate the prognosis of the visual symptoms [4]. Importantly, we performed morphologic evaluations and quantitative measurements of the retina using OCT. Indeed, OCT allowed us to obtain precise measurements of retinal thickness. However, as discussed above, OCT is fundamentally unsuitable for evaluating horizontal shift of the retina. Furthermore, it is difficult to evaluate the disarray of the outer segment of each photoreceptor cell using OCT in view of its relatively lower resolution in the retinal plane [26].

Despite these shortcomings, we performed quantitative measurements of metamorphopsia associated with acute CRVO using M-CHARTS scores. Even after reduction of ME, more than half of our patients still suffer from decreased quality of vision due to metamorphopsia. Further prospective studies with longer follow-up periods are necessary to elucidate the long-term changes in metamorphopsia associated with CRVO.

## Supporting information

S1 File Specific dataset for all individuals. (XLSX)
